# Driving sustainable change: A systematic map of behaviorally informed interventions to promote sustainable mobility behavior

**DOI:** 10.1093/pnasnexus/pgaf162

**Published:** 2025-05-22

**Authors:** Michael Bissel, Maike Gossen, Lucia A Reisch, Cass R Sunstein

**Affiliations:** Technische Universität Berlin, Straße des 17. Juni 135, Berlin 10623, Germany; Technische Universität Berlin, Straße des 17. Juni 135, Berlin 10623, Germany; University of Cambridge, El-Erian Institute for Behavioural Economics and Policy, Cambridge Judge Business School, Trumpington St 25, Cambridge CB2I1 AG, United Kingdom; Harvard University, 1563 Massachusetts Avenue, Cambridge, MA 02138, USA

**Keywords:** choice architecture, nudge, behavior change, evidence synthesis, sustainable mobility

## Abstract

Recent reports have highlighted the importance of changing human behavior if climate change is to be mitigated. In this respect, behaviorally informed interventions are considered promising tools. In particular, these interventions could be applied in the transport sector, where the mitigation potential is high. However, a comprehensive synthesis of the evidence on behaviorally informed interventions in this field is lacking, although such syntheses are extremely useful for researchers, policymakers, and funding bodies alike. This study addresses this gap by presenting a systematic map of behaviorally informed interventions that target sustainable mobility decisions. An extensive review of more than 30,000 articles revealed a substantial corpus of 204 relevant studies. While studies on usage behavior (e.g. fuel-efficient driving) and studies addressing the private context are most prevalent, the synthesis highlights that existing studies investigated a remarkably diverse set of heterogeneous mobility decisions. Additionally, studies addressing the professional context or positioned at the intersection of the private and professional sphere were identified, highlighting the potential for behavior change interventions in multiple contexts. This study provides a foundational resource for understanding the scope of existing research and uncovering underexplored areas with high mitigation potential. The findings not only inform future academic research but also guide policymakers and practitioners in designing effective, behaviorally informed strategies to reduce transportation's environmental impact.

**Significance Statement**

Evidence syntheses, such as systematic maps, are an indispensable resource for researchers, policymakers, and funders. Previous syntheses have indicated the potential value of behaviorally informed interventions as a complement to traditional policy instruments in numerous domains. Unfortunately, evidence syntheses in the transport sector are limited even though, for that sector, behaviorally informed interventions are promising in view of the sector's overall carbon emissions, the mitigation potential of related individual decisions, and heuristics and biases affecting those decisions. Therefore, this study addresses the need for a comprehensive systematic map of behaviorally informed interventions in the context of sustainable mobility, offering an overview of the existing research. Based on the analysis, implications for future research are discussed.

## Introduction

Climate change is a consequence of human behavior and therefore requires significant adjustments in individual and collective behavior if it is to be successfully combatted (1, 2). The latest report from the Intergovernmental Panel on Climate Change highlights the pivotal role of demand-side behavioral change strategies in achieving emission reduction targets (3). This need is particularly pronounced in the transport sector, a major source of emissions and a potential “roadblock” to climate change mitigation (4, 5).

In this context, the potential of behaviorally informed modifications to the choice architecture has attracted attention as a means of nudging consumers to arrive at certain decisions (6). Such nudges can be described as a “GPS system” that guides individuals in a certain direction and at the same time gives them the opportunity to choose alternative options (7). In line with this metaphor, the transport sector represents a promising area of application for behaviorally informed interventions (8).

Despite this potential, previous studies suggest that the transport sector has received relatively little attention in research on behaviorally informed interventions compared with other domains (9, 10). For instance, flagship reports by international organizations (11, 12) and major academic evidence syntheses (2, 13) have not separately addressed the transport sector in their analyses of behaviorally informed interventions.

Moreover, existing reviews that include individual mobility decisions and behaviorally informed interventions have notable shortcomings. Many cover only isolated decisions such as mode choice (14, 15), do not systematically distinguish between specific decisions (10, 16), or are limited to mobility in the private context (14). In addition, reviews often do not use established taxonomies to categorize interventions or do not focus specifically on behaviorally informed interventions (17–19). Methodologically, some reviews rely on a limited number of databases or exclusively on peer-reviewed articles (10, 19). Taken together, these shortcomings lead to a high risk of the evidence base being underestimated and relevant studies ignored. Thus, while some existing reviews are characterized by a considerable level of detail, a comprehensive evidence synthesis covering the full range of mobility-related outcomes and different types of behaviorally informed interventions is currently lacking.

Comprehensive evidence syntheses, however, are of significant value to policymakers, researchers, and funding bodies. Some types of evidence syntheses, such as meta-analyses, focus on answering specific questions, often related to one particular intervention or outcome. Other types of synthesis, while characterized by the same level of methodological rigor and transparency, have a broader scope (20–22). Systematic maps, in particular, are especially well-suited for collating, cataloging, and describing evidence on broad, heterogeneous topics and outcomes (20, 22–24).

As demonstrated for behaviorally informed interventions in other domains (24, 25), systematic maps provide valuable insights with regard to, inter alia, existing research clusters that are suitable for subsequent meta-analyses, research gaps that require further primary research, and existing evidence that is relevant for policymakers (20–22). Despite this potential, there appears to be no comprehensive systematic map on behaviorally informed interventions in the context of sustainable mobility. This article addresses this research gap by presenting what, to the best of the authors' knowledge, is the most extensive systematic map on this topic to date.

### Behaviorally informed interventions

The term behaviorally informed interventions describes small, low-cost, choice-preserving, and low-intrusion “nudges” that are informed by findings from cognitive psychology and behavioral economics (6, 24). These academic disciplines extend economic models by taking empirical findings on human behavior into account (6). As such, behaviorally informed interventions provide a cost-effective and citizen-centered complement to the traditional policy toolkit (16, 24, 26, 27). Since the early 2010s, there has been a notable surge in research and applications in the field of behavioral public policy (28, 29). This surge is reflected in the increasing number of academic articles that refer to nudging (2) and the global spread of behavioral public policy bodies that translate those concepts into practice (30).

Prior empirical research and syntheses have assessed behaviorally informed interventions in numerous policy domains such as health, nutrition, finance, and environment (2, 15, 24, 27). In the context of sustainability, these interventions are regarded as a promising approach in view of the obstructive characteristics of decisions such as intertemporality, uncertainty, and conflicting interests (31–33).

Behaviorally informed interventions encompass both educational and architectural tools (34). While numerous frameworks exist to categorize specific techniques, Münscher et al. (35) have developed a taxonomy of choice architecture tools that focuses on intervention design rather than on underlying cognitive processes and is therefore particularly suitable for cataloging empirically tested interventions (2, 10, 15). This taxonomy identifies three main categories with nine specific techniques: decision information (e.g. providing feedback or information on norms), decision structure (e.g. adjusting defaults or option-related effort), and decision assistance (e.g. providing reminders or facilitating commitment) (35).

### Mobility as a promising area of application for behaviorally informed interventions

Globally, the transport sector accounts for approximately one-quarter of energy-related greenhouse gas emissions, with emissions growing faster than in any other end-use sector (5, 36). At the same time, physical mobility remains a fundamental aspect of individual and societal functioning and is referred to as the “blood system” of modern societies (37). This dual role—critical contributor of emissions and key enabler of social activities—highlights the need for innovative policy approaches that reduce the sector's environmental impact without undermining its core benefits.

Compared with other sectors, the transport sector offers considerable opportunities to reduce emissions through individual behavioral changes (38, 39). Unlike other domains (2), mobility decisions span a wide temporal spectrum, encompassing short-term behaviors and long-term decisions (40). More precisely, a comprehensive framework for mobility decisions categorizes decisions into five distinct types (41): (i) upstream decisions, long-term choices such as residential location or vehicle ownership; (ii) travel decisions (whether, where, and how often to travel); (iii) route and mode choice; (iv) usage behavior (e.g. fuel-efficient driving and carbon offsetting); and (v) downstream decisions regarding existing possessions, such as reducing car ownership. The framework also differentiates between the private and the professional context, recognizing that mobility decisions often occur within or are influenced by organizational settings (e.g. business travel, professional driving, and commuting). In particular, as discussed in more detail in the description of the framework (41), interventions in the professional context can be implemented in a more direct and targeted manner and can take advantage of organizational norms and dynamics (41–43). In addition, professional contexts are often characterized by different financial incentives which can lead to principal-agent problems (41, 44).

In addition, both short- and long-term mobility decisions are subject to various heuristics and biases and thus are not always fully rational (8, 40, 45). For example, short-term decisions such as mode choice have been found to be influenced by habits, norms, status seeking, and the status quo effect. While these aspects are also relevant to long-term decisions such as car purchase, this type of decision is also influenced by bounded rationality in terms of mental accounting and linearity heuristics (40). For example, the miles per gallon illusion demonstrates how individuals often misinterpret fuel efficiency metrics, leading to suboptimal vehicle choices (46). Since behaviorally informed interventions, in contrast to traditional policy instruments, explicitly address such phenomena, they can facilitate more sustainable mobility decisions (8).

### The present study

In light of the above findings, this study provides a systematic map that is comprehensive in terms of mobility-related outcomes, contexts, and search strategies while focusing on behaviorally informed interventions. An extensive range of outcomes and contexts reflects the numerous interdependent entry points for interventions in the mobility context (41). In alignment with the overarching objectives of systematic maps (21), the study seeks to identify existing research foci, general patterns, and research gaps pertaining to, among other things, the investigated outcomes and interventions. To this end, it investigates the following research question: Which behaviorally informed interventions have been studied that target different types of mobility decisions by individuals in private and professional contexts with the aim of reducing the negative environmental impact of transportation?

For the investigation, the study followed a rigorous and transparent evidence synthesis process based on a preregistered review protocol (47). The analyses are based on established frameworks for categorizing studies. Specifically, the contextual mobility decisions matrix developed by Bissel et al. (41) is used to cover the full range of mobility decisions. In addition, the taxonomy of choice architecture techniques by Münscher et al. (35) serves as a basis for classifying interventions. While systematic maps do not evaluate the effectiveness of interventions (22), this study does approximate the mitigation potential of targeted outcomes based on an existing meta-analysis (39).

The following section summarizes the results of the evidence synthesis in a general description of the evidence base followed by summaries regarding outcomes, decision context, mitigation potential, interventions, as well as study types and methodology. On that basis, the discussion section starts with a summary of the results before providing implications for future empirical research, for further evidence syntheses, and for practitioners. The “Materials and methods” section then summarizes the methodological approach. Further information on the methodology is given in the SI Appendix.

## Results

Based on a comprehensive literature search and screening strategy that covered bibliographic databases and search engines, 28,950 articles were retrieved. After removing duplicates, 16,624 entries remained for screening. Following screening based on predefined criteria and multiple supplementary search strategies that retrieved additional articles, 204 individual studies were included in the analysis (see Fig. S1 for a ROSES flow diagram).

### Description of the evidence base

**Fig. 1.**
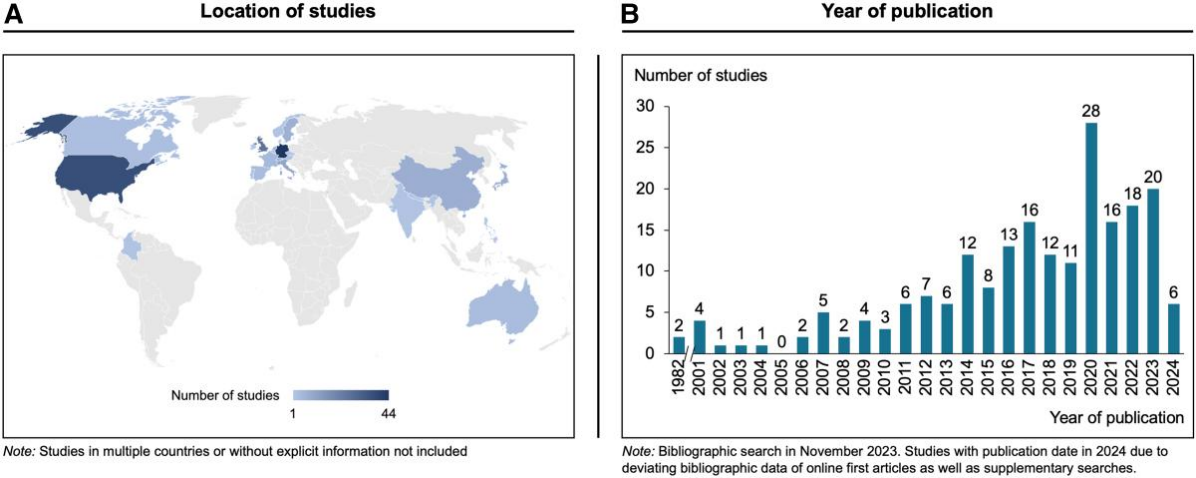
Geographical and temporal spread of included studies.

Figure 1 provides an overview of the geographical distribution and years of publication of the included studies. The evidence base comprises studies conducted in 26 countries (see Table S8), indicating a broad geographical distribution. However, slightly more than half (52%) of the studies originate from three countries: Germany, the United States, and the United Kingdom (see Fig. 1A).

Figure 1B shows generally a steady rise in research over the past decades. The evidence base dates back to pioneering studies conducted in the 1980s on behaviorally informed interventions to encourage public transport use (48, 49) and reaches a peak in 2020. The data highlight a significant increase in interest over the past decade, with 73% of all included studies published in 2015 or later.

### Outcomes

**Fig. 2.**
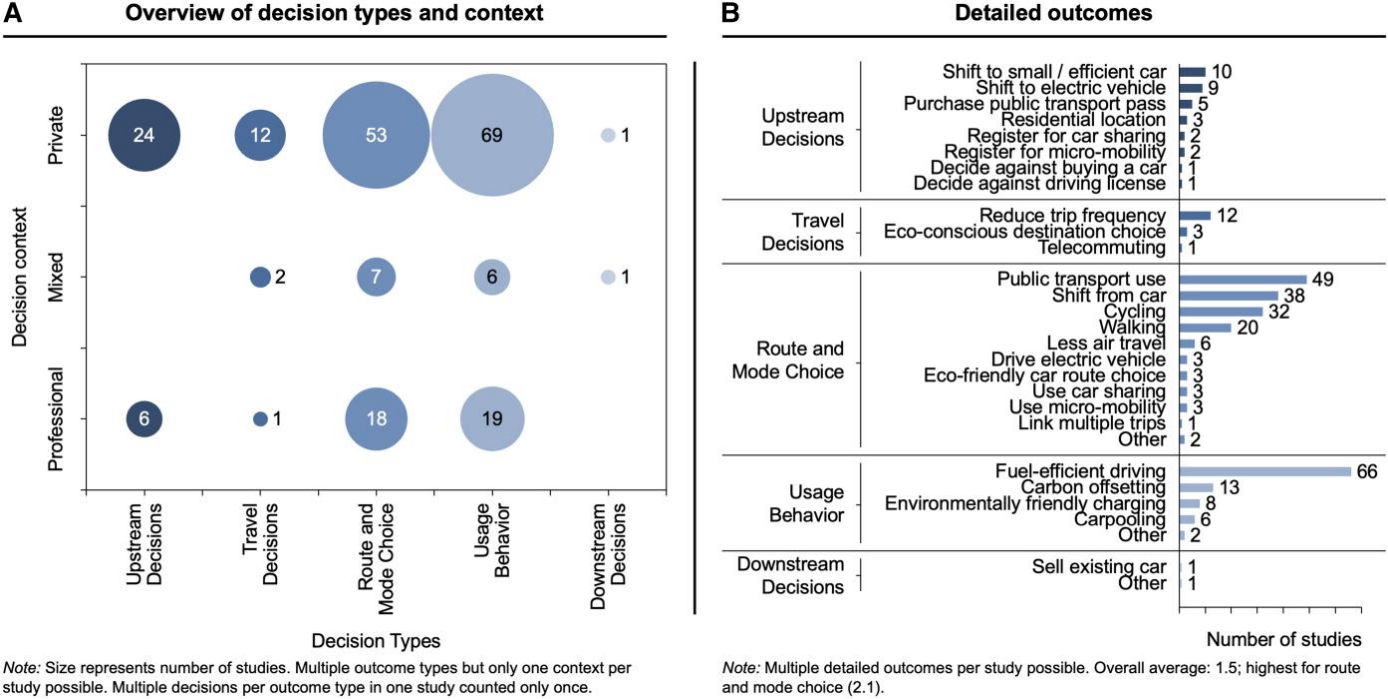
Overview of decision types and context A) and detailed outcomes B) of included studies.

As illustrated in the overview in Fig. 2A, the evidence synthesis revealed empirical trials across all decision types. The majority of studies address usage behavior (46%), followed by route and mode choice (38%), upstream decisions (15%), and travel decisions (7%). Downstream decisions are investigated in only two studies (50, 51). Several studies targeted multiple outcome types. A closer examination of the detailed outcomes underscores the broad and heterogeneous set of targeted decisions that are included in the overarching decision types (Fig. 2B).

While usage behavior, the most prevalent decision type, is dominated by a substantial body of research on fuel-efficient driving (52–56), the investigated outcomes extend far beyond driving in the narrow sense. They include subtypes of fuel-efficient driving such as checking tire pressure (57) and stopping idling (58, 59). In addition, in the context of electric vehicles, interventions to shift charging demand to optimize renewable energy use were investigated (60–62).

Upstream decisions, as another example, comprise a wide range of low-frequency decisions that provide important preconditions for medium- and short-term mobility decisions (41). The evidence synthesis revealed studies that aimed to reduce the need for individualized motorized travel by facilitating transport-optimized residential choices (63–65). Other interventions aimed at encouraging car-free living by addressing the option of forgoing car purchases (66) or promoting deliberate decisions on whether or not to acquire a driving license (67). In addition to not owning a car, studies tested behaviorally informed interventions to promote electric vehicle adoption (68, 69), a one-time decision that can substantially reduce the lifetime carbon emissions of car ownership (39). Another area of research focused on expanding mobility options by setting necessary preconditions for environmentally friendly modes of transportation, such as acquiring public transport season tickets (70), registering for bike-sharing (71), or adopting micromobility solutions (72).

### Decision context

The majority of interventions were tested in the private context (74%). In addition, a notable portion of the evidence base comprises studies that employed interventions in the professional context (21%). Moreover, 6% of all studies were positioned at the intersection of the professional and private context (“Mixed” category in Fig. 2A) (41).

While the private context has also been analyzed in previous reviews (14), a detailed analysis of the present evidence base reveals nuanced insights. For example, included studies tested interventions that target both daily mobility (73–75) and vacation decisions (76, 77). In addition, periods of transition in the private context were strategically leveraged to change mobility behavior (78).

In the professional context, existing studies addressed the ecodriving behavior of truck drivers in Australia (79), Colombia (80), and Germany (81, 82). In addition, postal delivery (83) and taxi drivers (84, 85) were investigated. Other studies addressed the adoption of public transport job tickets (86) or employees' decisions for electric company cars (43). Regarding air travel, studies investigated academic conference travel (87, 88) and even the ecofriendly behavior of airline captains (89). A range of studies also addressed environmentally friendly commuting (90–96).

Studies in the “mixed” category include, for example, interventions that are implemented in the professional context but whose effects are also measured in the private context. Exemplary studies targeted the environmentally friendly use of mobility budgets (97) or ecodriving with company cars in the private and professional context (98).

### Mitigation potential

**Fig. 3.**
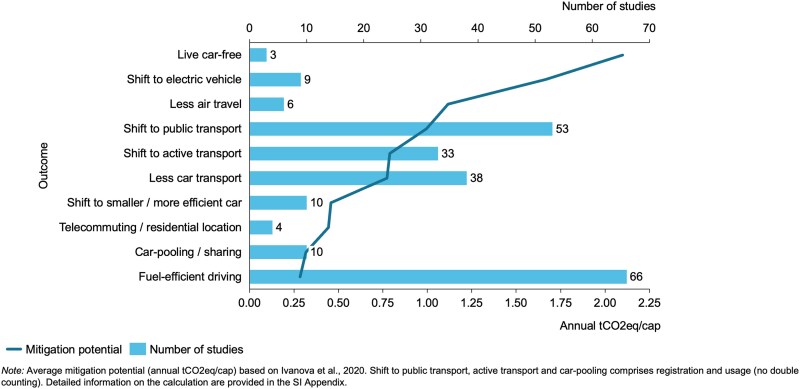
Mitigation potential of the outcomes addressed in included studies.

The number of studies investigating specific outcomes can be mapped on the average mitigation potential per capita and year of specific decisions as categorized and synthesized in a meta-analysis by Ivanova et al. (39). As illustrated in Fig. 3, the outcomes addressed by the studies in the evidence base predominantly target decisions with medium to low mitigation potential. In contrast, high-impact decisions such as living car-free, shifting to an electric vehicle, or reducing air travel are studied to a lesser extent.

### Interventions

**Fig. 4.**
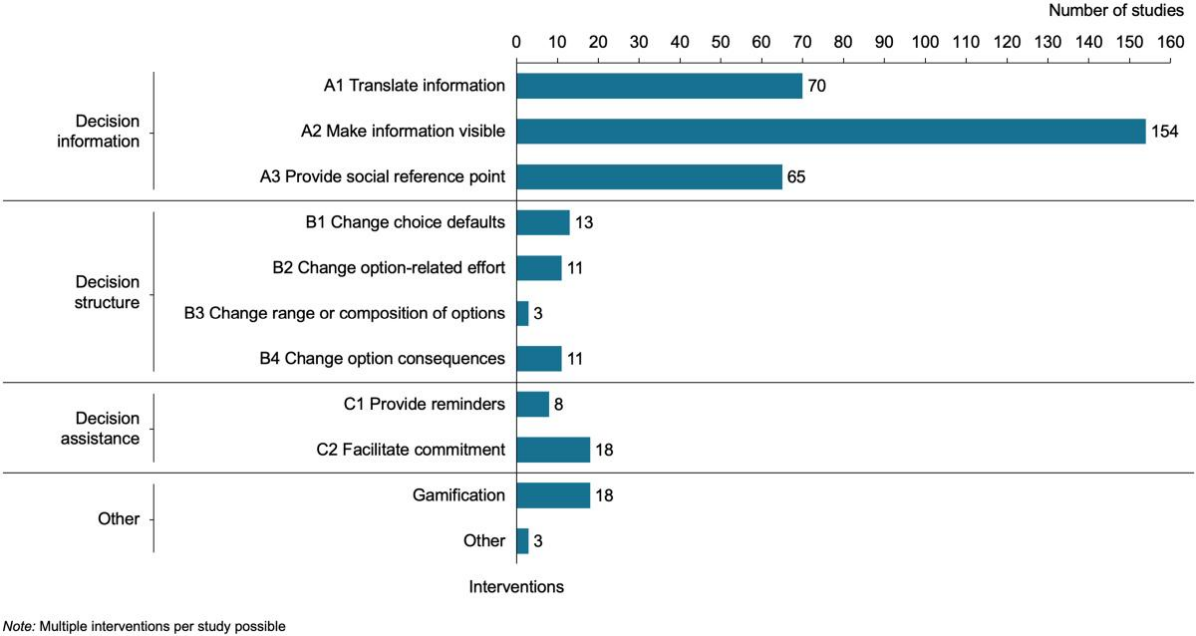
Interventions tested in included studies. Taxonomy based on Münscher et al. (35).

Figure 4 provides an overview of the prevalence of the different types of choice architecture interventions in the evidence base. As can be seen in the figure, decision information is the dominant category in the evidence base. In line with the significant proportion of decision information interventions, the vast majority of all studies (86%) included an educational component. For example, car labels, which represent a frequently discussed policy example, were tested (99). In addition to the three categories proposed by Münscher et al. (35), interventions in 18 studies featured gamification elements.

Besides this general overview, an in-depth analysis of tested interventions by outcome type shows that decision information was used most frequently across all outcome types and that the relative share of such interventions (e.g. feedback) is particularly high for usage behaviors such as fuel-efficient driving. The highest share of the intervention types decision structure and decision assistance was found in the “route and mode choice” outcome type (see Fig. S2).

More than half of the included studies (56%) employ a combination of multiple intervention techniques. For example, such studies provide feedback and translate it into easily understandable indicators (61). Only 16% of the studies test combinations with traditional policy instruments. Examples of such combinations are those with economic incentives (51, 100, 101) and regulations such as separated lanes for high-occupancy vehicles (102). Other studies combine behaviorally informed interventions with new services (103).

### Study types and methodology

Randomized controlled trials represent the most commonly used study design (38%), followed by survey-based experiments (27%) and other forms of field experiments (20%). Fifteen percent of the studies were conducted as laboratory experiments.

In terms of sample size, 34% of studies are based on samples of <100 participants while 38% use a sample size between 100 and 500 participants and 25% use more than 500 participants. The remaining 3% of studies are based on study designs without a defined sample size.

Moreover, 70% of all studies assess actual behaviors, while the remaining 30% of studies rely on hypothetical choices and intentions. Notably, the proportion of hypothetical decisions is considerably higher for long-term decisions such as upstream decisions than for short-term decisions (see Fig. S3).

The risk of bias assessment (see “Materials and methods”) leads to an aggregated overview of the threats related to internal validity (see Fig. S4). The assessment indicates a considerable risk of bias in reporting and methodology: 34 and 25% of all studies provided insufficient information on randomization and group allocation concealment, respectively. Furthermore, a pronounced risk of bias was found in terms of performance (55%) and detection bias (44%), which can be primarily attributed to inadequate blinding of participants combined with the frequent use of self-reported outcome measures.

## Discussion

The objective of this systematic map was to provide a comprehensive overview of behaviorally informed interventions in the context of sustainable mobility.

### Summary of main results

The study reveals a considerable evidence base with 204 included studies. This scope of evidence is notable in comparison with other domains (15, 24) and particularly in view of previous reviews that have suggested limited empirical research on behaviorally informed interventions in the transport context (9, 10).

The fact that this systematic map could be based on such an extensive corpus of evidence may be driven by several factors. The present study covers a wide range of outcomes and contexts. Moreover, the exhaustive search strategy, particularly the supplementary search steps such as snowball sampling, resulted in the screening of a broad scope of literature from multiple disciplines. In addition, the analysis of publication years shows that research has recently increased significantly, underlining the value of up-to-date evidence syntheses. This remarkable increase in research seems plausible given the growing interest in behaviorally informed interventions (2, 24) and the increasing concern about rising emissions in the transport sector (5).

Although the evidence is drawn from a wide range of countries, the focus is on three Western nations: Germany, the United States, and the United Kingdom. This finding corresponds to the pronounced interest in behavioral public policy (24, 30) and the particularly pressing environmental consequences of transport in those countries (104).

The present study indicates that existing research focuses on usage behavior such as fuel-efficient driving. At the same time, the map reveals a remarkably extensive set of investigated outcomes. Thus, irrespective of the proportion of these studies, this finding underscores the numerous and diverse opportunities for behavior change that go beyond altering short-term behavior.

In terms of mitigation potential, the investigated outcomes are associated with relatively medium to low mitigation potential. However, the present study provides grounds for a less pessimistic conclusion regarding coverage of high-impact behaviors than that provided in a previous review on consumer research (105).

The included studies predominantly tested decision information techniques. This is particularly notable given previous studies that have emphasized the potential and effectiveness of another category of interventions, namely decision structure interventions such as defaults (2, 32, 106). Additionally, less than one in five of all studies tested combinations with nonbehaviorally informed interventions. Although this relatively low proportion might be partly due to the strict inclusion criteria of this study (see Table S5), such combinations are considered promising both within and outside the transportation sector (8, 16, 45, 107).

In terms of methodology, the high prevalence of randomized controlled trials is consistent with expectations, as this study type is the traditional gold standard for intervention testing (8, 28). Furthermore, the majority of studies assess actual behaviors as outcomes. In particular, the higher proportion of hypothetical decisions in long-term decisions could potentially comprise the external validity of those studies (24) and limit comparability between decision types.

### Implications for future research

The systematic map developed in this study points to several direct implications for future research. As it provides a comprehensive overview of existing evidence, in line with the objectives of systematic maps (20, 21), it can be utilized to identify targets for subsequent systematic reviews. For example, route and mode choice and usage behavior are potential clusters with sufficient evidence that could be the subject of future meta-analyses. Furthermore, a review of habitual decisions, such as usage behavior, compared with long-term, high-impact decisions could provide important insights into which interventions work best for which decision types. With regard to the decision context, a distinct analysis of intervention effectiveness in the professional context could provide valuable insights, particularly in view of potential spill-over effects to the private context and organizations' capability to implement direct interventions (41, 43).

Moreover, the identified research gaps can inform the design of future empirical studies. These research gaps especially relate to high-impact decisions such as living car-free, switching to electric vehicles, or reducing air travel. In particular, downstream decisions related to existing possessions represent a significant avenue for future research given the mitigation potential and the role of status quo biases (41, 108). This study also underscores the need for additional research into the effectiveness of interventions targeting decision structures or providing decision assistance. Furthermore, future research should focus on combining behaviorally informed interventions with traditional policy instruments such as economic incentives (16) and integrating behavioral public policy with other fields such as urban planning (109). The geographical overview of the existing evidence base reveals a need for further research outside Europe and North America, in particular in developing regions, where travel-related emissions have increased most rapidly in recent years and are expected to continue to do so in the future (5). Analyzing intervention effectiveness in different geographical settings would be beneficial (8). Doing so would also complement previous comparative studies on intervention acceptance (110).

Future studies should also give due consideration to the methodological aspects summarized in this map. When assessing outcomes, more studies should focus on evaluating actual behaviors rather than hypothetical choices, especially for long-term decisions. This focus may require new forms of transdisciplinary collaboration between researchers and nonacademic stakeholders (111). The evidence base provides several illustrative examples of successful collaboration between researchers and external organizations. These examples include car dealerships (112) and manufacturers (43), public transport providers and municipalities (78, 113) as well as airlines (89) and airports (94). In addition, interdisciplinary collaboration is recommended to increase the effectiveness of behaviorally informed interventions (16). For example, interdisciplinary projects involving behavioral scientists and computer scientists could be mutually beneficial as they enable technologically sophisticated digital interventions that are explicitly based on behavior change theories. These suggestions, in combination with more transparent reporting and more rigorous methodology (10), would also lead to a reduction of the considerable risks of bias which were identified as part of the critical appraisal of internal validity.

While this study covers a variety of mobility-related outcome types and contexts, several studies that did not meet the inclusion criteria provide suggestions for future systematic maps. For example, adjacent behaviors were addressed, including proenvironmental hotel and tourist choices (114, 115), e-commerce and deliveries (116, 117), or time-of-use electricity tariffs (118). Moreover, some excluded studies have explored decisions that could have indirect environmental effects, for instance, the orderly use and redistribution of shared vehicles (119–122), the reduction in dwell times at subway stations (123), or the prevention of bicycle theft (124). Finally, future evidence syntheses could focus on additional roles such as members of organizations (e.g. fleet managers) or citizens (e.g. policy acceptance) (125). An analysis of these roles would further underscore the complexity of the challenges at hand and the necessity of individual and collective action (1).

### Implications for practitioners and policy

The evidence collated in this systematic map, in conjunction with the searchable database of primary studies, has the potential to inform policymakers and other practitioners regarding potential interventions. In particular, the evidence base is distinguished by the diversity of included outcomes. Because the professional context was included, the evidence base is also pertinent to employers seeking to develop effective interventions for their staff. To meet the specific information needs of practitioners and policy makers, the searchable database includes a number of additional variables, including geographic context. The analyses presented here therefore provide an overarching overview of the most important dimensions, in line with the objectives of systematic maps. While the systematic map does not focus on the effectiveness of interventions, the breadth of outcomes, interventions, and approaches provides a first indication of the potential of behaviorally informed interventions in the context of sustainable mobility.

### Limitations

Although the present study adheres to established methodological standards and guidelines, some limitations should be noted. As is inevitable due to the logic of the search process, the retrieval of studies from bibliographic databases and search engines depends on whether the search terms are used by the authors of the primary studies. This limitation was addressed by the iterative development of the search strings and supplementary search steps. While studies were screened regardless of the original language of the full text, documents would not have been retrieved in this search step if an English abstract was not available. However, this limitation was also mitigated by the comprehensive supplementary search steps (see “Materials and methods”). In terms of data extraction, the framework for mobility decisions that was used (41) provides a detailed coding system for relevant outcomes. However, the granularity of the systematic map depends on the level of detail of the outcome reporting in the primary studies. Lastly, in line with the recommendations for systematic maps, the critical appraisal of the present study is limited to a risk of bias assessment of internal validity. For subsequent systematic reviews, a more detailed critical appraisal is required, including an assessment of external validity (22).

## Materials and methods

The present evidence synthesis was conducted in accordance with the guidelines of the Collaboration for Environmental Evidence (20) and follows the ROSES reporting standards (126).

### Search strategy

Six bibliographic databases were included: Scopus, Web of Science Core Collection, PsycInfo, Business Source Complete, Academic Search Premier, and Social Science Premium Collection. In line with recommendations on identifying relevant gray literature to reduce the risk of publication bias (127), a search engine (Google Scholar) and a database for dissertations and theses (ProQuest Dissertations and Theses) were included in the search. To ensure comprehensive coverage, no date restrictions or other filters were applied. These searches were conducted in November 2023.

A detailed search string was developed for searching the bibliographic databases and the repository for dissertations and theses (see Table S1). This search string was constructed by combining search terms relating to the interventions (e.g. choice architecture), the outcomes (e.g. individual mobility decisions), and the framing (e.g. climate change). The search string was pretested against a test list of articles developed by the authors and then iteratively refined, taking into account specificity and sensitivity (20). In line with the specifications of the respective databases, the search string was adapted and relevant search fields were selected (see Table S2).

To complement this primary search, additional steps were taken to ensure comprehensive coverage, as proposed for systematic maps (22). These steps included screening the references of all included studies from the primary search process (backward snowballing) and all articles citing the respective articles (forward snowballing). For all articles listed in Scopus, this process resulted in an additional 9,561 entries to be screened. In addition, 34 organizational websites were screened based on an updated and refined list from a previous study (24) (see Table S3). Moreover, 40 existing systematic reviews and reports were consulted (see Table S4). From those sources, 1,004 articles were extracted for screening. Finally, several experts were consulted directly and via a survey published on an institutional social media site (22).

### Screening criteria and process

All retrieved articles were subjected to a screening process based on six predefined eligibility criteria. The criteria were based on the “PICO-FS” logic (24), which represents an extension of the widely used “PICO” criteria (20). This process ensured that all included articles fell within the scope of the research question of this systematic map (see Table S5). To ensure comprehensive coverage, no language restrictions were applied.

Screening was performed manually for the entire set of references. Two authors conducted the screening at title, abstract, and full-text level, as described in the ROSES flowchart (see Fig. S1). To assess interrater reliability, a random sample of 10% of all articles retrieved from bibliographic databases and search engines was analyzed (24). Cohen's kappa indicated substantial (0.69 at the title level; 0.74 at the abstract level) to almost perfect (0.87 at the full-text level) consistencies between raters based on the criteria proposed by Landis and Koch (128). Any remaining conflicts were discussed between raters until consensus was reached.

### Data extraction

The relevant data for the systematic map were extracted from the original documents using a predefined data extraction template (see Table S6). When extracting intervention and outcome data, multiple coding was possible to enable accurate representation of complex studies. Two authors performed data extraction separately. Consistency was checked using a 10% sample of all included studies. Conflicts were discussed until consensus was reached.

### Critical appraisal

Although critical appraisal is optional for systematic maps, a risk of bias assessment was conducted (20–22, 126). In accordance with Frampton et al. (129), the Cochrane Collaboration's tool for assessing risk of bias (130) was selected for critical appraisal due to its widespread use, which includes similar reviews (131). As stated in the preregistration (47) and suggested by guidelines for systematic maps (22), this assessment was solely conducted to inform the overall evaluation of methodological rigor and to derive implications for future research. Therefore, no studies were excluded based on the critical appraisal.

## Supplementary Material

pgaf162_Supplementary_Data

## Data Availability

Data to replicate the analysis will be made publicly available via OSF repository: https://osf.io/j56s8/.
