# Supplementary material for: Driving sustainable change: A systematic map of behaviorally informed interventions to promote sustainable mobility behavior
Source: PNAS Nexus. 2025 May 22;4(6):pgaf162. doi: 10.1093/pnasnexus/pgaf162 (PMC12147027; doi:10.1093/pnasnexus/pgaf162)
Supplement: pgaf162_Supplementary_Data [file pgaf162_supplementary_data.zip › 250405_PNAS_Nexus_SI_Appendix_DrivingSustainableChange.pdf]

PNAS nexus

Supporting Information for
Driving sustainable change: A systematic map of behaviorally informed interventions to promote sustainable mobility behavior

Michael Bissel[a,*], Maike Gossen[a], Lucia A. Reisch[b], Cass R. Sunstein[c]

[a] Technische Universität Berlin, Straße des 17. Juni 135, 10623 Berlin, Germany

[b] University of Cambridge, El-Erian Institute for Behavioural Economics and Policy, Cambridge Judge Business School, Trumpington St 25, Cambridge CB2 I1AG, United Kingdom

[c] Robert Walmsley University Professor, Harvard University, 1563 Massachusetts Avenue, Cambridge, MA, 02138, USA

*Corresponding author: Michael Bissel

Email: michael.bissel@campus.tu-berlin.de

This PDF file includes:

Supporting text
Figures S1 to S4
Tables S1 to S8
SI References

Other supporting materials for this manuscript include the following:

Datasets (S1)

Supporting Information Text

Materials and Methods

Search strategy. The overarching search strategy was developed using the Collaboration for Environmental Evidence (1) guidelines. In addition, the ROSES reporting standards were followed (2). A corresponding flow diagram is shown in Fig. S 1. The selection of databases and the development of the search string were informed by previous evidence syntheses (3–9). In addition, an experienced librarian from Harvard University provided guidance as a search mentor. Following guidelines for systematic maps (10), the search strategy was complemented with several supplementary search steps: screening of organizational websites, screening of existing literature reviews, snowball sampling, expert consultations, and a social media survey. Prior to commencing the search process, the study underwent ethical review and received approval by the Departmental Ethics Review Group at Cambridge Judge Business School (approval reference 23-35).

Bibliographic search and search engines. The iterative search string development is described in detail in the supplementary material to the preregistered review protocol (11). As stated in the preregistration (12), a simplified search string was used for Google Scholar, due to technical limitations of the search engine (8). Additionally, screening in this search engine was limited to the first 1,000 entries (13), with separate searches for British and American spelling. Table S 1 provides an overview of the resulting search strings. The searches in bibliographic databases and search engines were conducted on November 25, 2023. Table S 2 summarizes the searches and number of retrieved articles in each database.

Supplementary search strategies. The supplementary search was particularly designed to retrieve grey literature, which describes articles not formally published by academic publishers, such as academic theses, organizational reports, and government papers (14).

The list of organizational websites (see Table S 3) was updated from a previous systematic map of behaviorally informed interventions (8) and adapted to the context of this study to include leading organizations in relevant fields (behavioral science, sustainability, and / or mobility). Organizational websites were searched using keywords for intervention (nudge, nudging, choice architecture, behavioral public policy, behavioral insights, behavioral change, behavioral economics, behavioral intervention) and outcome (mobility, transport, travel) based on the Google Scholar search string (see Table S 1). Searches were conducted in English or with the German equivalents, depending on the primary language of the website.

Articles from existing literature reviews and reports (see Table S 4) were extracted from supplementary material, reference lists, or full texts. Wherever possible, extraction was restricted to relevant outcomes or interventions.

Snowball sampling included screening references cited in included studies (backward snowballing) and articles citing these articles (forward snowballing). For articles listed in Scopus (71%), references were exported and screened in the same way as articles from bibliographic databases. References of the remaining articles were screened manually and Google Scholar was used for forward snowballing.

The supplementary search strategies were completed in June 2024 (organizational website screening, existing literature reviews) and September 2024 (snowball sampling), respectively. Datasets for forward snowballing were exported from Scopus in July 2024. Relevant documents from the supplementary search stages were added to the full-text screening stage as additional pre-screened articles.

Screening. All entries retrieved from the bibliographic databases were exported and added to an EndNote library. For Google Scholar, a specialized tool was used for this purpose (15). All articles were checked for duplicates using EndNote 21. After deduplication, all remaining references were imported into the online platform Rayyan, which was used to facilitate the screening process. All articles were screened based on PICO-FS criteria (8),

which are summarized for this study in Table S 5. These criteria were ranked and applied based on (presumed) ease of finding information: outcome, intervention, framing, population, study type, comparator. Screening was performed separately at the title, abstract, and full text level. Two authors (M.B. and M.G.) manually screened the retrieved articles. Each article was reviewed by at least one reviewer. The assignment of reviewers for the screening process ensured that members of the project team did not decide on the eligibility of their own authored articles.

Full texts were retrieved using library subscriptions from Technische Universität Berlin, University of Cambridge, Leuphana Universität Lüneburg, and Harvard University. Articles not available through these subscriptions were requested via interlibrary loans or directly from the authors. A list of articles that were not retrievable after these steps is provided in the supplementary data. While articles were searched using English and (for relevant organizational websites) German search terms, articles in all languages were screened for eligibility to minimize a selective bias toward the Global North. For this purpose, articles in languages other than English or German were translated using a professional translation software.

Data extraction. The data extraction template was created based on recommended templates (1) and previous research (8). This extraction template included bibliographic information, information about the inclusion criteria, and other information about the study (see Table S 6). Data extraction was performed using the SRDR+ online platform.

Mitigation potential. To approximate the mitigation potential of the outcomes addressed, the studies included in this systematic map were mapped to the average mitigation potential of specific individual decisions in the transport sector as synthesized by Ivanova et al. (5). A comparison of the categories and corresponding outcomes in the systematic map is provided in Table S 7.

Results

The supplementary tables and figures in this SI appendix provide additional information and complementary insights into the reported results. Table S 8 provides a complete list of countries in which studies were conducted and the number of studies for each country. Fig. S 2 provides a detailed analysis of the distribution of intervention types by outcome types. Fig. S 3 illustrates the proportion of actual versus hypothetical outcomes by outcome type. Finally, Fig. S 4 shows the aggregated results of the critical appraisal.

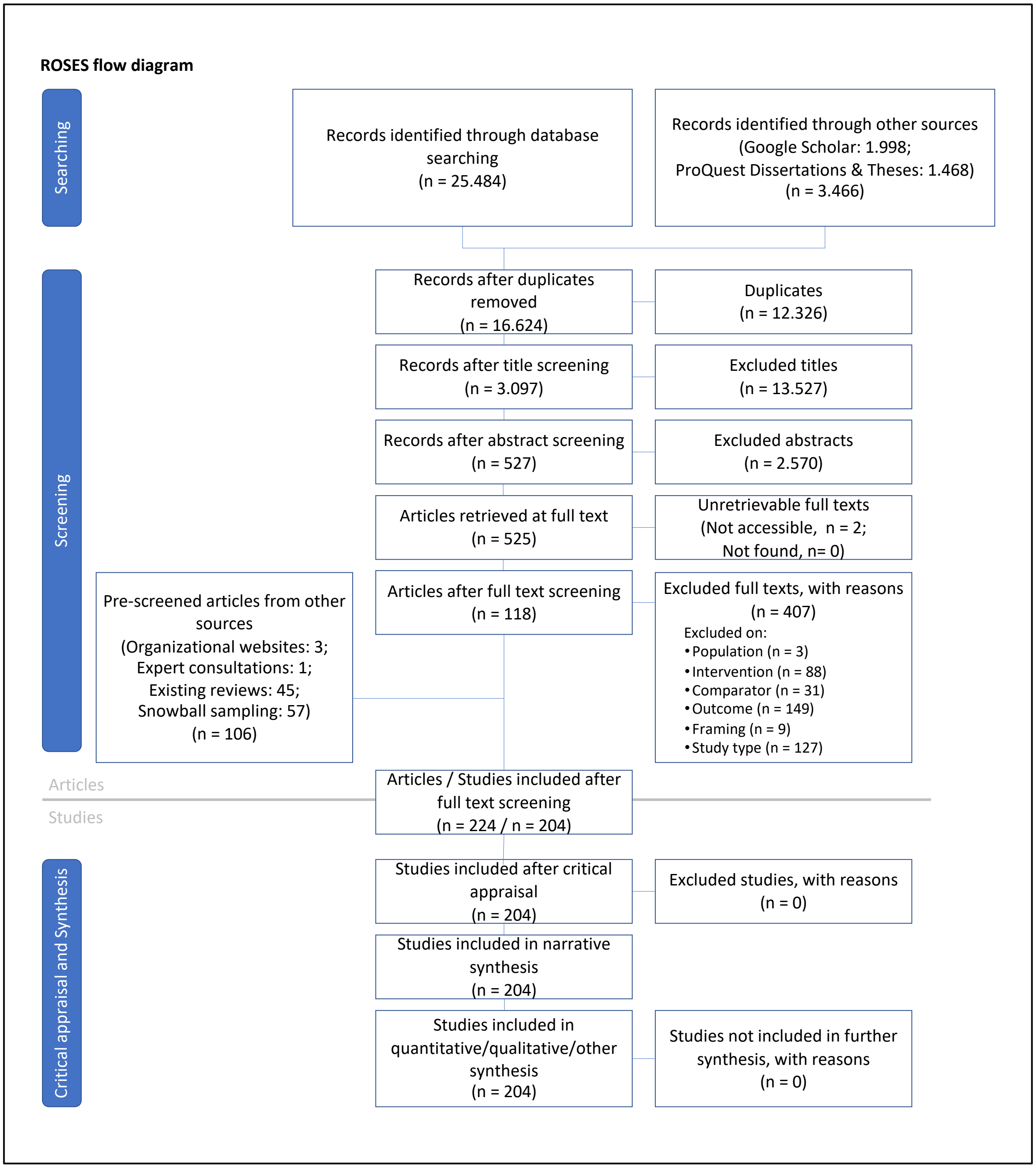

Fig. S 1. ROSES flow diagram

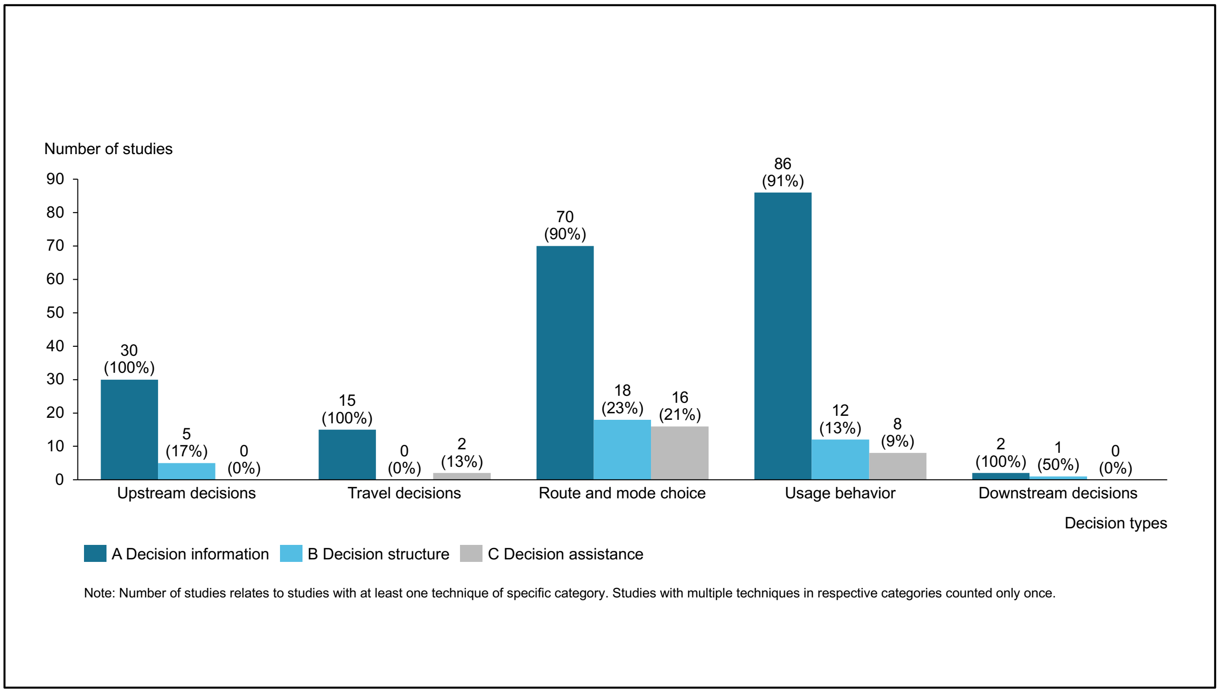

Fig. S 2. Interventions and outcome types

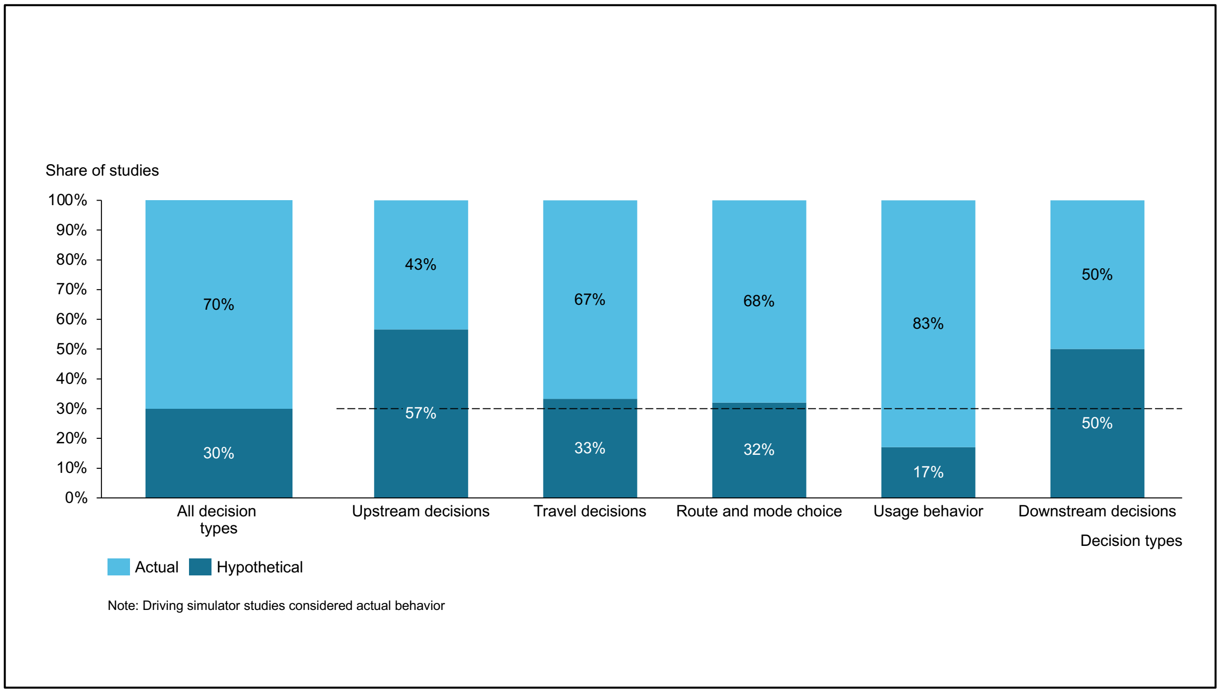

Fig. S 3. Outcome measurement by outcome type

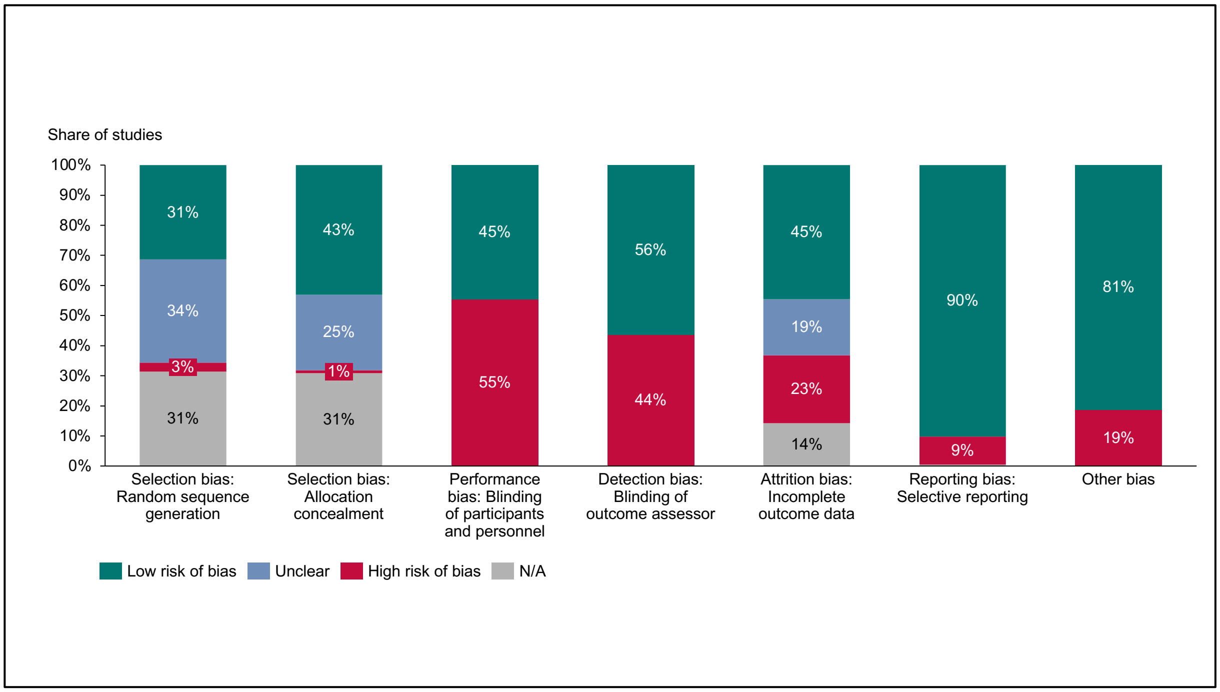

Fig. S 4. Overview of Critical Appraisal

Table S 1. Search strings

Full boolean search string (Scopus version)	TITLE-ABS-KEY((nudg* OR "choice architecture" OR "behavior* public policy" OR "behavior* economics" OR "behavior* insights" OR "behavior* intervention*" OR "behavior* stimul*" OR (behavior* W/2 chang*)) AND (mobil* OR transport* OR travel* OR trip OR touris* OR transit OR destination OR route OR "mode choice" OR automobile* OR vehicl* OR car OR bik* OR bicycl* OR train OR bus OR subway OR underground OR metro OR walk* OR airplan* OR plane* OR driv* OR rid* OR charg* OR fly* OR flight* OR offset* OR "home office" OR remote OR commut* OR telecommut* OR telework* OR residential* OR hous* OR employment* OR job*) AND (((atmospheric OR anthropogenic OR effect* OR emission* OR footprint* OR mitigat* OR sav* OR reduc* OR budget* OR impact* OR decreas*) AND (carbon OR CO2 OR "greenhouse gas*" OR GHG OR GHGs)) OR (climat* AND (action* OR chang* OR warm* OR shift*)) OR ((energy OR fuel) AND (efficien* OR consum* OR use)) OR sustainab* OR combustion OR pollution OR "global warming" OR "emission reduction*" OR (mitigation AND (action* OR potential*))))
Short search string (Google Scholar)	(nudge OR nudging OR “choice architecture” OR "behavioral public policy" OR "behavioral insights" OR “behavioral change” OR “behavioral economics” OR “behavioral intervention”) AND (mobility OR transport OR travel) AND ("climate change" OR "greenhouse gas" OR emissions OR sustainability)

Table S 2. Overview of bibliographic databases

No.	Database	Platform	Field codes	Retrieved articles
1	Scopus	Scopus	Title, abstract, keywords	8,957
2	Web of Science Core Collection	Web of Science	Title, abstract, author keywords, keywords plus	7,799
3	PsycInfo	Ebsco	Title, abstract, keywords, subjects, authors	1,169
4	Business Source Complete	Ebsco	Title, abstract, keywords, subjects, authors	1,600
5	Academic Search Premier	Ebsco	Title, abstract, keywords, subjects, authors	3,975
6	Social Science Premium Collection	ProQuest	Anywhere except full text	1,984

Table S 3. Overview of organizational websites

No.	Name	Geography	Link
1	Behaven	BE	https://behaven.com/en
2	Behavioural Insights Team	Global	www.bi.team/
3	Behavioral Science and Policy Association	Global	https://behavioralpolicy.org
4	BehaviourWorks Australia,	AUS	https://www.behaviourworksaustralia.org
5	BETA - Behavioural Economics Team of the Australian Government	AUS	https://behaviouraleconomics.pmc.gov.au
6	ConPolicy	GER	https://www.conpolicy.de
7	Department for Environment, Food & Rural Affairs	UK	https://www.gov.uk/government/organisations/department-for-environment-food-rural-affairs
8	Department for Transport	UK	https://www.gov.uk/government/organisations/department-for-transport
9	EIT Urban Mobility	EU	https://www.eiturbanmobility.eu/
10	Environment Agency	UK	www.gov.uk/government/organisations/environment-agency
11	Environmental Protection Agency	USA	www.epa.gov/
12	European Commission Joint Research Centre	EU	www.ec.europa.eu/jrc/en
13	European Environment Agency	EU	www.eea.europa.eu/
14	Federal Environment Agency	GER	www.umweltbundesamt.de/
15	Federal Ministry for the Environment, Nature Conservation, Nuclear Safety and Consumer Protection	GER	https://www.bmuv.de
16	Federal Ministry for Digital and Transport	GER	https://bmdv.bund.de/
17	Harvard Kennedy School Shorenstein Center on Media, Politics and Public Policy, Behavioral Insights Group	US	https://shorensteincenter.org/programs/behavioral-insights-group/
18	International Energy Agency	Global	https://www.iea.org
19	National Platform Future of Mobility	GER	https://www.plattform-zukunft-mobilitaet.de/en/
20	Organisation for Economic Co-operation and Development	Global	www.oecd.org/

21	OPSI - Observatory of Public Sector Innovation	Global	https://oecd-opsi.org
22	PBL Netherlands Environmental Assessment Agency	NL	www.pbl.nl/en/
23	Princeton University, Kahneman-Treisman Center for Behavioral Science & Public Policy	US	https://behavioralpolicy.princeton.edu
24	Technical University of Munich, MCube	GER	https://www.mcube-cluster.de/en/
25	The London School of Economics and Political Sciences (LSE), LSE Cities	UK	https://www.lse.ac.uk/cities
26	The World Bank	Global	www.worldbank.org/
27	Travalyst	Global	https://travalyst.org/about/
28	United Nations Environment Programme	Global	www.unenvironment.org/
29	United Nations Framework Convention on Climate Change	Global	www.unfccc.int/
30	United Nations Human Settlements Programme,	Global	https://unhabitat.org
31	United Nations Innovation Network	Global	https://www.uninnovation.network
32	University of St. Gallen – Institute for Mobility	CH	https://imo.unisg.ch/de/home/
33	University of Toronto, Behavioural Economics in Action at Rotman	CA	www.rotman.utoronto.ca/FacultyAndResearch/ResearchCentres/BEAR
34	Nudgd.io	SE	https://nudgd.io

Note: Organization 8, 21, and 34 added to initial preregistered list

Table S 4. List of screened literature reviews and reports

No.	Reference	Retrieved articles
1	Arnott, B., Rehackova, L., Errington, L., Sniehotta, F. F., Roberts, J., & Araujo-Soares, V. (2014). Efficacy of behavioural interventions for transport behaviour change: Systematic review, meta-analysis and intervention coding. International Journal of Behavioral Nutrition and Physical Activity,	13
2	Composto, J. W., & Weber, E. U. (2022). Effectiveness of behavioural interventions to reduce household energy demand: A scoping review. *Environmental Research*	43
3	Creutzig, F., Callaghan, M., Ramakrishnan, A., Javaid, A., Niamir, L., Minx, J., Müller-Hansen, F., Sovacool, B., Afroz, Z., Andor, M., Antal, M., Court, V., Das, N., Díaz-José, J., Döbbe, F., Figueroa, M. J., Gouldson, A., Haberl, H., Hook, A., … Wilson, C. (2021). Reviewing the scope and thematic focus of 100 000 publications on energy consumption, services and social aspects of climate change: A big data approach to demand-side mitigation *. Environmental Research Letters, 16(3), 033001. https://doi.org/10.1088/1748-9326/abd78b	40
4	Forberger, S., Reisch, L., Kampfmann, T., & Zeeb, H. (2019). Nudging to move: A scoping review of the use of choice architecture interventions to promote physical activity in the general population. International Journal of Behavioral Nutrition and Physical Activity, 16(1), 77. https://doi.org/10.1186/s12966-019-0844-z	5
5	Lehner, M., Mont, O., & Heiskanen, E. (2016). Nudging – A promising tool for sustainable consumption behaviour? Journal of Cleaner Production, 134, 166–177. https://doi.org/10.1016/j.jclepro.2015.11.086	6
6	Möser, G., & Bamberg, S. (2008). The effectiveness of soft transport policy measures: A critical assessment and meta-analysis of empirical evidence. Journal of Environmental Psychology, 28(1), 10–26. https://doi.org/10.1016/j.jenvp.2007.09.001	5
7	Pawluk De-Toledo, K., O'Hern, S., & Koppel, S. (2022). Travel behaviour change research: A scientometric review and content analysis. Travel Behaviour and Society, 28, 141–154. https://doi.org/10.1016/j.tbs.2022.03.004	17
8	Szaszi, B., Palinkas, A., Palfi, B., Szollosi, A., & Aczel, B. (2018). A Systematic Scoping Review of the Choice Architecture Movement: Toward Understanding When and Why Nudges Work: Systematic Scoping Review of the Nudge Movement. Journal of Behavioral Decision Making, 31(3), 355–366. https://doi.org/10.1002/bdm.2035	4
9	Aneeque Javaid, Tarun Khanna, Mercedes Franza et al. Behavioural interventions change individual transport choices but have a limited impact on transport mode split. Evidence from a systematic review, 02 November 2022, PREPRINT (Version 1) available at Research Square [https://doi.org/10.21203/rs.3.rs-2084989/v1]	288
10	Macmillan, A. K., Hosking, J., L. Connor, J., Bullen, C., & Ameratunga, S. (2013). A Cochrane systematic review of the effectiveness of organisational travel plans: Improving the evidence base for transport decisions. Transport Policy, 29, 249–256. https://doi.org/10.1016/j.tranpol.2012.06.019	12
11	Riggs, W. (2022). Behavioral economics and social nudges in sustainable travel. In Innovations in Transport (pp. 89–110). Scopus. https://www.scopus.com/inward/record.uri?eid=2-s2.0-85160884323&partnerID=40&md5=65d0ea61837aa333ab22dca1067504d3	23

12	Zimmermann, Sina & Hein, Andreas & Schulz, Thomas & Gewald, Heiko & Krcmar, Helmut. (2021). Digital Nudging Toward Pro-Environmental Behavior: A Literature Review Completed Research Paper.	43
13	Kormos, C., Sussman, R., & Rosenberg, B. (2021). *How Cities Can Apply Behavioral Science to Promote Public Transportation use*.	15
14	Casquero, D., Monzon, A., García, M., & Martínez, O. (2022). Key Elements of Mobility Apps for Improving Urban Travel Patterns: A Literature Review. Future Transportation, 2(1), 1–23. https://doi.org/10.3390/futuretransp2010001	54
15	Thorun, C., Diels, J., Vetter, M., Reisch, L., Bernauer, M., Micklitz, H.-W., Rosenow, J., Forster, D., & Sunstein, C. R. (2017). Nudge-Ansätze beim nachhaltigen Konsum: Ermittlung und Entwicklung von Maßnahmen zum „Anstoßen" nachhaltiger Konsummuster. Umweltbundesamt.	6
16	Whillans, A., Sherlock, J., Roberts, J., O'Flaherty, S., Gavin, L., Dykstra, H., & Daly, M. (2021). Nudging the Commute: Using Behaviorally Informed Interventions to Promote Sustainable Transportation. Behavioral Science, 7(2).	11
17	Reindl, A., Juppe, M., Graf, P., & Putz-Egger, L.-M. (2023). The use of gamification to change commuters' mobility behavior: A literature review.	23
18	Anagnostopoulou, E., Bothos, E., Magoutas, B., Schrammel, J., & Mentzas, G. (2018). Persuasive Technologies for Sustainable Mobility: State of the Art and Emerging Trends. Sustainability, 10(7), 2128. https://doi.org/10.3390/su10072128	29
19	Bergram, K., Djokovic, M., Bezençon, V., & Holzer, A. (2022). The Digital Landscape of Nudging: A Systematic Literature Review of Empirical Research on Digital Nudges. CHI Conference on Human Factors in Computing Systems, 1–16. https://doi.org/10.1145/3491102.3517638	4
20	Graham-Rowe, E., Skippon, S., Gardner, B., & Abraham, C. (2011). Can we reduce car use and, if so, how? A review of available evidence. Transportation Research Part A: Policy and Practice, 45(5), 401–418. https://doi.org/10.1016/j.tra.2011.02.001	16
21	Carrel, C., Gavard-Perret, M.-L., & Caldara, C. (2023). Factors of effectiveness of green nudges for more eco-responsible behaviour – Systematic review and research directions. Recherche et Applications En Marketing (English Edition), 38(3), 32–76. https://doi.org/10.1177/20515707231177814	28
22	Fujii, S., & Taniguchi, A. (2006). Determinants of the effectiveness of travel feedback programs—A review of communicative mobility management measures for changing travel behaviour in Japan. Transport Policy, 13(5), 339–348. https://doi.org/10.1016/j.tranpol.2005.12.007	10
23	Wang, W., Gan, H., Wang, X., Lu, H., & Huang, Y. (2022). Initiatives and challenges in using gamification in transportation: A systematic mapping. European Transport Research Review, 14(1), 41. https://doi.org/10.1186/s12544-022-00567-w	30
24	Souza-Neto, V., Marques, O., Mayer, V. F., & Lohmann, G. (2023). Lowering the harm of tourist activities: A systematic literature review on nudges. Journal of Sustainable Tourism, 31(9), 2173–2194. https://doi.org/10.1080/09669582.2022.2036170	11
25	Wynes, S., Nicholas, K. A., Zhao, J., & Donner, S. D. (2018). Measuring what works: Quantifying greenhouse gas emission reductions of behavioural interventions to reduce driving, meat consumption, and household energy use. Environmental Research Letters, 13(11), 113002. https://doi.org/10.1088/1748-9326/aae5d7	2
26	Byerly, H., Balmford, A., Ferraro, P. J., Hammond Wagner, C., Palchak, E., Polasky, S., Ricketts, T. H., Schwartz, A. J., & Fisher, B. (2018). Nudging pro-	15

environmental behavior: Evidence and opportunities. Frontiers in Ecology and the Environment, 16(3), 159–168. https://doi.org/10.1002/fee.1777

27	Hermsen, S., Frost, J., Renes, R. J., & Kerkhof, P. (2016). Using feedback through digital technology to disrupt and change habitual behavior: A critical review of current literature. Computers in Human Behavior, 57, 61–74. https://doi.org/10.1016/j.chb.2015.12.023	4
28	Münsch, M., & Lell, O. (2024). Anreize zur Förderung eines nachhaltigen Mobilitätsverhaltens. Stand der Forschung zu Wirkung und Einsatzmöglichkeiten materieller, immaterieller und spielerischer Anreize. Umweltbundesamt.	113
29	Lembregts, C., & Cadario, R. (2024). Consumer-driven climate mitigation: Exploring barriers and solutions in studying higher mitigation potential behaviors. International Journal of Research in Marketing, S016781162400020X. https://doi.org/10.1016/j.ijresmar.2024.04.001	8
30	European Commission. (2016). Behavioural insights applied to policy: European report 2016. Publications Office. https://data.europa.eu/doi/10.2760/903938	16
31	OECD. (2017). Behavioural Insights and Public Policy: Lessons from Around the World. OECD. https://doi.org/10.1787/9789264270480-en	1
32	Aibana, K.; Kimmel, J.; Welch, S. (2017). Consuming Differently, Consuming Sustainably: Behavioural Insights for Policymaking.	5
33	de Hallgren, S. C., & Root-Bernstein, M. (2018). Changing Behaviours, Changing Policy – Evidence on Behavioural Insights for Green Growth.	4
34	Booth, S., Cavatassi, R., Curtis, B., Kim, D. S., Kim, Y., Langer, L., Mapitsa, C. B., Mokgano, E., Nduku, P., Puri, J., & Robertsen, J. (2022). Evidence review on behavioural interventions in development and environmental fields in developing countries.	3
35	IEA. (2020). Behavioural insights for demand-side energy policy and programmes: An environment scan. International Energy Agency. https://doi.org/10.47568/6OR105	27
36	Varazzani, C., Sullivan-Paul, M., & Tuomaila, H. (2023). Behavioural science for sustainable tourism: Insights and policy considerations for greener tourism (OECD Working Papers on Public Governance 60; OECD Working Papers on Public Governance, Vol. 60). https://doi.org/10.1787/c2ec4fcf-en	6
37	Bird, E. L., Baker, G., Mutrie, N., Ogilvie, D., Sahlqvist, S., Powell, J., & iConnect Consortium. (2013). Behavior change techniques used to promote walking and cycling: A systematic review. Health Psychology, 32(8), 829–838. https://doi.org/10.1037/a0032078	33
38	Sanguinetti, A., Queen, E., Yee, C., and Akanesuvan, K. 2020. "Average Impact and Important Features of Onboard Eco-Driving Feedback: A Meta-Analysis," Transportation Research Part F: Traffic Psychology and Behaviour (70), pp. 1-14.	13
39	Nisa, C. F., Bélanger, J. J., Schumpe, B. M., & Faller, D. G. (2019). Meta-analysis of randomised controlled trials testing behavioural interventions to promote household action on climate change. Nature Communications, 10(1), 4545. https://doi.org/10.1038/s41467-019-12457-2	16
40	Decrinis, L., & Reisch, L. A. (2023). Nudging employees for corporate sustainability: A systematic evidence map. In C. R. Sunstein & L. A. Reisch (Eds.), Research Handbook on Nudges and Society (pp. 152–173). Edward Elgar Publishing. https://doi.org/10.4337/9781035303038.00017	2

Table S 5. Inclusion and exclusion criteria

Question component	Eligibility criteria
Population (P): Individuals in different contexts such as in their role as private consumers and users or as working professionals with any form of work-related travel	*Include:* Individuals in their role as private persons (i.e., vehicle / public transport users; consumers regarding the purchase of vehicles or public transport tickets) or working professionals (e.g., full time driver or job-related travel); general population or specific subgroups (e.g., students)<br><br>*Exclude:* Institutions; individuals in their role as citizens, public decision makers, corporate decision makers, city planners
Intervention (I): Behaviorally informed interventions such as nudges or choice architecture	*Include:* Interventions that can be classified as behaviorally informed interventions[1] (e.g., nudge or choice architecture); educative and architectural nudges; interventions initiated by public, private or public-private institutions; interventions tested by researchers in hypothetical situations; interventions that are combined with other (otherwise excluded) interventions are only included if the intervention's effect can be separated<br><br>*Exclude:* Interventions that restrict freedom of choice in any form (e.g., bans); interventions that substantially change economic incentive structures (e.g., taxes, subsidies), other interventions that cannot be classified as behaviorally informed interventions
Comparator (C): Interventions have been studied which implies some form of comparison against a control group or a before-after comparison	*Include:* Effect is compared with a control group (no intervention or alternative intervention) or with another point in time (before – after); other forms of within-subjects designs also included<br><br>*Exclude:* Studies without any form of comparison
Outcome (O): Different types of mobility behaviors (i.e., upstream decisions, travel decisions, route and mode choice, usage behavior, downstream decisions)	*Include:* Different mobility behaviors and decisions with regard to upstream decisions (e.g. purchase of electric vehicle; including residential or employment choices if they are explicitly related to mobility decisions), travel decisions, route and mode choice, usage behavior or downstream decisions (e.g., selling a vehicle); actual or hypothetical behavior or stated behavioral intentions<br><br>*Exclude:* Any behavior that is not related to mobility; attitude, interest or preferences regarding behavior or transport modes
Framing (F): Interventions are introduced for environmental or climate purposes (e.g., reducing GHG emissions)	*Include:* Any form of reference to ecological sustainability, sustainable mobility, GHG emission reduction, fuel savings or energy savings; sustainability to be mentioned in article but not necessarily in the intervention context; environmental benefits should at least be explicitly mentioned - however, might be secondary focus besides other framing (e.g., health); to be classified as relevant, climate framing must be established early in the paper (that is, before the discussion and conclusion section)<br><br>*Exclude:* Other framings such as exclusive focus on health (e.g., by increasing physical activity) or safety (e.g., by avoiding speeding); commercial outputs (e.g., shared mobility providers aiming to promote services) - if not combined with environmental framing
Study types (S): Interventions are studied with any form of empirical method	*Include:* All kinds of study designs with some form of empirical methodology (quantitative or qualitative) that provide primary data (e.g., experimental studies, panel studies, interview studies, observational studies)<br><br>*Exclude:* All sorts of studies without empirical methodology and without primary data (e.g., conceptual papers, methodological descriptions, reviews, commentaries or similar studies). Studies that do not introduce / observe interventions but rather measure (perceived) levels (e.g., social norms).

Note: 1. Based on the taxonomy provided by Münscher et al. (16)

Table S 6. Meta-data extraction and coding sheet

Category	Type of data	Meta-data / coding
Bibliographic information[2]	a) Year of Publication	Meta-data
	b) Publication type	Codes: peer-reviewed article, book chapter, conference proceedings, report / white paper, thesis / dissertation, other
Information relating to the inclusion criteria	a) Population: context	Codes: private, professional, mixed[1]
	b) Population: geographic setting	Codes: urban, rural, n/a
	c) Population: description	Meta-data
	d) Intervention: typology element	A1 translate information; A2 make information visible; A3 provide social reference point; B1 change choice defaults; B2 change option-related effort; B3 change range or composition of options; B4 change option consequences; C1 provide reminders; C2 facilitate commitment; gamification[1]; other
	e) Intervention: description	Meta-data
	f) Intervention: educative element	Yes, No
	g) Intervention: non-behavioral combination	Yes, No
	h) Comparator: general	Codes: Control, Pre/Post, Mixed[1], other
	i) Comparator: description	Meta-data
	j) Outcome: decision	Codes: upstream decisions, travel decisions, route and mode choice, usage behavior, downstream decisions, other
	k) Outcome: details	Residential location, employment location, decide against buying a car, decide against driving license, shift to EV, shift to small / efficient car, acquire bicycle, purchase public transport pass, register for car-pooling / sharing, register for micro-mobility, reduce trip frequency, eco-conscious destination choice, telecommuting, drive EV, eco-friendly car route choice, link multiple trips, less air travel, use car-pooling / sharing, use micro-mobility, public transport use, cycling, walking, fuel efficient driving, environmentally friendly charging, carbon off-setting, sell existing car, prolong use of existing car, other
	l) Outcome: type	Codes: actual, hypothetical
	m) Outcome: description	Meta-data
	n) Framing: climate framing (keywords used)	Meta-data
	o) Framing: GHG change measured	Yes, No
	p) Framing: Percentage of GHG reduction	Meta-data
	q) Framing: first climate mentioning (page)	Meta-data
	r) Framing: additional framing	Codes: health, safety, both, none, other
	s) Study type: design	Codes: field experiment (RCT), field experiment (other), lab experiment, survey-based experiment, survey, other
	t) Study type: description	Meta-data
Information related to the study	a) Study duration	Meta-data
	b) Sample size	Codes: <100; 100-500; 500-1,000; 1,000-3,000; 3,000 – 5,000; 5,000-10,000; >10,000
	c) Study: Location	Meta-data

Note: 1. Codes added to preregistered form; 2. Basic bibliographic information also included

Table S 7. Approximation of mitigation potential

Category	Ivanova et al.[1]	Outcomes in systematic map[2]	Mitigation potential[3]
Live car-free	Original category "Live car-free" (aggregated consumption option).	"Decide against buying a car", "Decide against driving license", "Sell existing car"	2.10
Shift to electric vehicle	Aggregated consumption options "Shift to BEV" and "Shift to PHEV/HEV".	"Shift to EV"	1.67
Less air travel	Aggregated consumption options "one less flight (long return)", "one less flight (medium return)" and "less transport by air".	"Less air travel"	1.12
Shift to public transport	Original category "Shift to public transport" (aggregated consumption option)	"Purchase public transport pass", "public transport use"	0.99
Shift to active transport	Original category "Shift to active transport" (aggregated consumption option)	"Walking", "Cycling", "Acquire bicycle"	0.79
Less car transport	Original category "Less car transport" (aggregated consumption option)	"Shift from car"	0.77
Shift to smaller / more efficient car	Aggregated consumption options "Shift to a smaller car" and "energy and material efficiency" in consumption domain "Transport"	"Shift to small / efficient car"	0.46
Telecommuting / residential location	Original category "Telecommuting" (aggregated consumption option), which includes original consumption options such as "live closer to workplace"	"Telecommuting", "Residential location", "Employment location"	0.44
Car-pooling / sharing	Original category "Car-pooling/sharing" (aggregated consumption domain)	"Register for car sharing", "use car sharing", "carpooling"	0.32
Fuel-efficient driving	Original category "Fuel efficient driving" (aggregated consumption domain)	"Fuel efficient driving"	0.28

Note: 1. from supplementary material; 2. Each study counted only once; 3. Annual tCO2eq per capita

Table S 8. Geographical overview of studies

Country	Number of studies
Germany	44
United States	37
United Kingdom	26
Multiple or n/a	16
Italy	11
China	8
Japan	8
Switzerland	7
Sweden	7
Netherlands	6
France	5
Spain	4
Australia	4
Canada	3
Colombia	2
Norway	2
Austria	2
Belgium	2
India	2
Portugal	1
Czech Republic	1
Denmark	1
Philippines	1
Israel	1
Nepal	1
Ireland	1
Singapore	1

SI References

1. Collaboration for Environmental Evidence, "Guidelines and Standards for Evidence synthesis in Environmental Management. Version 5.1" (2022).

2. N. R. Haddaway, B. Macura, P. Whaley, A. S. Pullin, ROSES RepOrting standards for Systematic Evidence Syntheses: pro forma, flow-diagram and descriptive summary of the plan and conduct of environmental systematic reviews and systematic maps. *Environ Evid* **7**, 7 (2018).

3. J. W. Composto, E. U. Weber, Effectiveness of behavioural interventions to reduce household energy demand: a scoping review. *Environ. Res. Lett.* **17**, 063005 (2022).

4. S. Forberger, L. Reisch, T. Kampfmann, H. Zeeb, Nudging to move: a scoping review of the use of choice architecture interventions to promote physical activity in the general population. *Int J Behav Nutr Phys Act* **16**, 77 (2019).

5. D. Ivanova, *et al.*, Quantifying the potential for climate change mitigation of consumption options. *Environ. Res. Lett.* **15**, 093001 (2020).

6. S. Mertens, M. Herberz, U. J. J. Hahnel, T. Brosch, The effectiveness of nudging: A meta-analysis of choice architecture interventions across behavioral domains. *Proc Natl Acad Sci USA* **119**, e2107346118 (2022).

7. K. Pawluk De-Toledo, S. O'Hern, S. Koppel, Travel behaviour change research: A scientometric review and content analysis. *Travel Behaviour and Society* **28**, 141–154 (2022).

8. L. A. Reisch, *et al.*, Mitigating climate change via food consumption and food waste: A systematic map of behavioral interventions. *Journal of Cleaner Production* **279**, 123717 (2021).

9. B. Szaszi, A. Palinkas, B. Palfi, A. Szollosi, B. Aczel, A Systematic Scoping Review of the Choice Architecture Movement: Toward Understanding When and Why Nudges Work: Systematic Scoping Review of the Nudge Movement. *J. Behav. Dec. Making* **31**, 355–366 (2018).

10. K. L. James, N. P. Randall, N. R. Haddaway, A methodology for systematic mapping in environmental sciences. *Environ Evid* **5**, 7 (2016).

11. M. Bissel, M. Gossen, S. Becker, L. A. Reisch, C. R. Sunstein, Search protocol for a systematic map of behaviorally informed interventions to promote sustainable mobility behavior - Supplementary Material 1: Search string development and databases. [Preprint] (2023). Available at: https://osf.io/y6qbc.

12. M. Bissel, M. Gossen, S. Becker, L. A. Reisch, C. R. Sunstein, Search protocol for a systematic map of behaviorally informed interventions to promote sustainable mobility behavior. [Preprint] (2023). Available at: https://osf.io/zrj8s.

13. N. R. Haddaway, A. M. Collins, D. Coughlin, S. Kirk, The Role of Google Scholar in Evidence Reviews and Its Applicability to Grey Literature Searching. *PLoS ONE* **10**, e0138237 (2015).

14. N. R. Haddaway, M. Land, B. Macura, “A little learning is a dangerous thing”: A call for better understanding of the term ‘systematic review.’ *Environment International* **99**, 356–360 (2017).

15. A.-W. Harzing, *The publish or perish book: your guide to effective and responsible citation analysis*, 1. ed (Tarma Software Research Pty Ltd, 2010).

16. R. Münscher, M. Vetter, T. Scheuerle, A Review and Taxonomy of Choice Architecture Techniques: Choice Architecture Techniques. *J. Behav. Dec. Making* **29**, 511–524 (2016).
